# Enhancement of Efficiency in an Ex Situ Coprecipitation Method for Superparamagnetic Bacterial Cellulose Hybrid Materials

**DOI:** 10.3390/membranes15070198

**Published:** 2025-07-01

**Authors:** Thaís Cavalcante de Souza, Italo José Batista Durval, Hugo Moraes Meira, Andréa Fernanda de Santana Costa, Eduardo Padrón Hernández, Attilio Converti, Glória Maria Vinhas, Leonie Asfora Sarubbo

**Affiliations:** 1 Center for Exact and Natural Sciences, Department of Materials Science, Federal University of Pernambuco (UFPE), Rua Prof. Moraes Rêgo 1235, Cidade Universitária, Recife 50670-901, Brazil; thsouza221@gmail.com (T.C.d.S.); eduardo.hernandez@ufpe.br (E.P.H.); 2 Advanced Institute of Technology and Innovation (IATI), Rua Potyra 31, Prado, Recife 50751-310, Brazil; italo.durval@iati.org.br (I.J.B.D.); hugo.meira@iati.org.br (H.M.M.); andrea.santana@ufpe.br (A.F.d.S.C.); 3 Department of Chemical Engineering, Federal University of Pernambuco (UFPE), Av. dos Economistas, Cidade Universitária, Recife 50740-590, Brazil; gloria.vinhas@ufpe.br; 4 Communication and Design Nucleus, Região Agreste Academic Center, Federal University of Pernambuco (UFPE), BR 104, km 59, s/n, Nova Caruaru, Caruaru 50670-901, Brazil; 5 Department of Physics, Federal University of Pernambuco (UFPE), Av. Jorn. Aníbal Fernandes s/n, Cidade Universitária, Recife 50740-540, Brazil; 6 Department of Civil, Chemical and Environmental Engineering, Pole of Chemical Engineering, Genoa University (UNIGE), Via Opera Pia 15, 16145 Genoa, Italy; converti@unige.it; 7 School of Technology and Communication, Catholic University of Pernambuco (UNICAP), Rua do Príncipe 526, Boa Vista, Recife 50050-900, Brazil

**Keywords:** bacterial cellulose (BC), magnetite nanoparticles, superparamagnetic biomembranes, superparamagnetism

## Abstract

Superparamagnetic magnetite nanoparticles (Fe_3_O_4_) have garnered considerable interest due to their unique magnetic properties and potential for integration into multifunctional biomaterials. In particular, their incorporation into bacterial cellulose (BC) matrices offers a promising route for developing sustainable and high-performance magnetic composites. Numerous studies have explored BC-magnetite systems; however, innovations combining ex situ coprecipitation synthesis within BC matrices, tailored reagent molar ratios, stirring protocols, and purification processes remain limited. This study aimed to optimize the ex situ coprecipitation method for synthesizing superparamagnetic magnetite nanoparticles embedded in BC membranes, focusing on enhancing particle stability and crystallinity. BC membranes containing varying concentrations of magnetite (40%, 50%, 60%, and 70%) were characterized using X-ray diffraction (XRD), Fourier-transform infrared spectroscopy (FTIR), scanning electron microscopy (SEM), and vibrating sample magnetometry (VSM). The resulting magnetic BC membranes demonstrated homogenous dispersion of nanoparticles, improved crystallite size (6.96 nm), and enhanced magnetic saturation (Ms) (50.4 emu/g), compared to previously reported methods. The adoption and synergistic optimization of synthesis parameters—unique to this study—conferred greater control over the physicochemical and magnetic properties of the composites. These findings position the optimized BC-magnetite nanocomposites as highly promising candidates for advanced applications, including electromagnetic interference (EMI) shielding, electronic devices, gas sensors, MRI contrast agents, and targeted drug delivery systems.

## 1. Introduction

Bacterial cellulose (BC) is a biopolymer produced by the fermentation of bacteria and microbial consortia. In addition to featuring nanometric-scale fibers and high hydrogel-forming ability, they can be easily produced without dependence on seasonal factors, large cultivation areas, or long and costly purification processes [1,2,3]. BC properties, such as the ability to absorb hydrophilic compounds when wet, biocompatibility, and strength, position it as a material with significant biotechnological potential for various applications, such as sensors, drug delivery systems, packaging, medical dressings, etc. [4,5]. Owing to all these characteristics and its production through renewable sources (corn steep liquor, tea, fruit residue. etc.), in numerous studies, BC-based materials have been widely used as matrices for producing hybrid and composite materials useful in fields such as medicine, pharmaceuticals, food, electronics, textiles, packaging, and environmental remediation [6,7,8,9].

New BC-based biomaterials with magnetic properties have shown promise as a biotechnological material in various studies, due to a wide range of synthetic routes and dopants for their production [10,11,12]. Additionally, there is a broad spectrum of applications, enabling these materials to be adapted to different manufacturing conditions of temperature, availability of reagents, and equipment to meet diverse demands [13,14,15].

Literature provides several examples of the application of magnetic bacterial cellulose-based materials, which have demonstrated excellent results. In the study by Maruthupandy et al. [16], for example, a composite of bacterial cellulose (BC) with graphene and magnetite was developed, showing strong antimicrobial activity against pathogenic bacteria such as *E. coli* and *P. mirabilis*. Similarly, in the work of Chaabane et al. [17], a drug delivery system based on magnetic BC demonstrated antimicrobial activity and exhibited antitumor effects in mice, effectively suppressing cancer development. In Chen et al. [18], a bacterial cellulose material with magnetite exhibited high efficiency in filtering an effluent containing an artificial coloring, Congo Red. In the field of sensors, Chen et al. [19] developed a flexible, wearable sensor composed of BC and cobalt ferrite, capable of monitoring motion. Fan et al. [20] created a material containing bacterial cellulose and magnetite with high shielding efficiency against electromagnetic waves. These examples highlight the variety and broad application potential of magnetic BC in diverse fields.

Magnetite (Fe_3_O_4_), which is one of the most commonly used dopants in producing these biomaterials, can exhibit either ferromagnetic or superparamagnetic characteristics, depending on its size [21,22,23,24,25,26]. Coprecipitation syntheses, both in situ and ex situ, are commonly employed for the production and incorporation of magnetite and other magnetic particles [27]. This methodology is widely used thanks to its simplicity and efficiency. However, adopting specific parameters influences the final size of particles and, consequently, their magnetic properties [28].

Souza et al. [29], who conducted a study on coprecipitation routes for in situ and ex-situ magnetite nanoparticles in BC membranes, observed that the method of incorporating magnetite influences the final size of the nanoparticles, the arrangement among BC fibers, and the magnetic properties. The results obtained were satisfactory; however, several studies suggest that some parameters could be optimized to obtain more stable superparamagnetic nanoparticles [30,31,32,33].

Based on the above, the aim of this study was to produce hybrid membranes of BC with superparamagnetic magnetite nanoparticles by enhancing the efficiency of the ex situ coprecipitation synthesis method.

## 2. Materials and Methods

### 2.1. Magnetic Biomembrane Production

#### 2.1.1. Microorganism Maintenance

The bacterium *Komagataeibacter hansenii* UCP1619, utilized for biocellulose (BC) production, was obtained from the Culture Bank of the Catholic University of Pernambuco (UNICAP), Recife, Brazil. The microorganism was preserved in HS medium, originally described by Hestrin and Schramm [34] and modified by Hungund and Gupta [35]. The medium consisted of 2.0% (*w*/*v*) glucose, 0.5% (*w*/*v*) yeast extract, 0.5% (*w*/*v*) peptone, 0.27% (*w*/*v*) disodium hydrogen phosphate (Na_2_HPO_4_), and 0.15% (*w*/*v*) citric acid (C_6_H_8_O_7_).

#### 2.1.2. Preparation of Pre-Inoculum

The procedure was carried out according to the methods described by Costa et al. [36] and Galdino et al. [37]. The bacterium was first activated and inoculated into HS agar medium and then incubated at 30 °C for 48 h. The activated cells were subsequently used to prepare the pre-inoculum, which was cultivated in liquid HS medium for 48 h at 30 °C.

#### 2.1.3. Biomembrane Production

For the fermentative production of BC, the production medium described by Costa et al. [36] and Galdino et al. [37] was used, a culture medium enriched with an agro-industrial residue, corn steep liquor, which enhances the productivity of BC membranes, reduces production costs, and provides an environmentally friendly use for a residue that would otherwise be discarded in nature. The medium consisted of 1.5% glucose, 2.5% corn steep liquor, 0.27% Na_2_HPO_4_, and 0.15% citric acid. After adjustment of the pH to 5, 3% of the pre-inoculum was added. BC biofilms were produced using a static fermentation method at 30 °C over a 10-day growth period. After production, the BC films were extracted from the fermented broth, cleaned, purified by immersion in a 4% NaOH solution for 2 h, and thoroughly washed with deionized water.

#### 2.1.4. Production of Magnetite Nanoparticles

The synthesis of magnetite via coprecipitation was optimized based on parameters found in literature, which favor the production of stable and superparamagnetic particles. The molar ratio of Fe^2+^ to Fe^3+^ was adjusted from 1:2 to 1:2.1, in accordance with studies by Goss [30], Jiang et al. [31], Eom et al. [32], and Shahid and Choi [33], which revealed that an increase in Fe^3+^ ions promotes the formation of magnetite while preventing the formation of maghemite. For this purpose, solutions of 3.329 mol/L FeCl_3_ and 1.96 mol/L FeCl_2_ were mixed in distilled water and vigorously stirred for 10 min. Adopting vigorous stirring led, in fact, to the increased reactivity of Fe^2+^ ions, thereby producing Fe_3_O_4_ particles with enhanced magnetic properties [38,39,40]. Subsequently, 100 g of NH_4_OH was slowly added, which turned the solution black, confirming a high percentage of magnetite formation by a qualitative observation. A process similar to that reported by Jiang et al. [31] was employed to purify and stabilize the synthesized particles. An ammonium oleate surfactant (composed of 15 g of oleic acid, 20 mL of distilled water, and 10 mL of NH_4_OH) was added to the product, forming micelles around the particles to protect them, and then an aqueous solution of hydrochloric acid (1:2 vol) was added to neutralize NH_4_OH and precipitate Fe_3_O_4_. The particles were finally collected with the aid of a magnet, washed with distilled water and isopropanol, and dried on a hot plate at 40 °C, thus preserving their magnetic properties. The Fe_3_O_4_ produced was named “MAG N”.

#### 2.1.5. Incorporation of Magnetite Nanoparticles into Biocellulose Membranes

Prior to incorporation, BC was processed using a processor, model New B (Mallory, São Paulo, Brazil), with a power output of 1000 W and a rotational speed of 16,750 rpm, resulting in a biocellulose paste. The use of a high-power, high-speed processor facilitated the processing of bacterial cellulose (BC) and contributed to the uniform dispersion of nanoparticles throughout the matrix. Subsequently, the nanoparticles were incorporated into the BC paste using the same equipment at magnetite concentrations of 40%, 50%, 60%, and 70%, generating the samples labeled BC40MAG, BC50MAG, BC60MAG, and BC70MAG, respectively.

The samples were molded in Petri dishes and dried in an oven at 40 °C for 24 h.

### 2.2. Physicochemical and Structural Characterizations

#### 2.2.1. X-Ray Diffraction (XRD)

XRD analysis was employed to identify the phase composition of the samples using an XRD-7000 diffractometer (Shimadzu, Kyoto, Japan). Measurements were conducted on powdered samples mounted on an aluminum support, covering a 2θ range from 10° to 80°, utilizing Cu Kα radiation (λ = 1.54056 Å) at a scanning rate of 0.5°/min in continuous mode. The crystallite sizes were determined by applying the Scherrer equation, following the methodology outlined by Burton et al. [41] and Mascolo et al. [42]. The crystallinity was calculated according to the methodology of Ruland and Vonk, as described in the study by Cheah and Masaharu [43].

#### 2.2.2. Fourier-Transform Infrared (FTIR) Spectroscopy

The identification of functional groups in the samples was carried out by FTIR spectroscopy using an IRTracer 100 spectrometer (Shimadzu, Kyoto, Japan). The spectra were recorded over a wavenumber range of 400 to 4000 cm^−1^, with a resolution of 4 cm^−1^ and an average of 128 scans.

#### 2.2.3. Scanning Electron Microscopy (SEM)

For SEM analysis, the samples were coated with a thin layer of gold using a sputtering technique. The imaging was performed at room temperature using a Mira 3 microscope (Tescan, Kohoutovice, Czech Republic). Micrographs were obtained at both micrometer and nanometer scales.

#### 2.2.4. Vibrating Sample Magnetometry (VSM)

Magnetization as a function of applied magnetic field (M vs. H) was measured at 300 K using a high-sensitivity magnetometer VSM (MicroSense model 3482-70 Electromagnet, Hamm, Germany). The magnetic field was applied within the range of −20 to 20 kOe, with the instrument operating at a sensitivity of 10^−5^ emu/g. The sample consisted of superparamagnetic magnetite (Fe_3_O_4_) nanoparticles, with a known density of 5.2 g/cm^3^. All experimental magnetization data were expressed in emu/g. The magnetic behavior of the superparamagnetic nanoparticles was analyzed using the Langevin function, a reliable method for estimating the size of Fe_3_O_4_ particles [44] that accurately describes non-interacting, single-domain particles with randomly oriented magnetic moments under an applied magnetic field. The Langevin model is given by Equation (1):

(1) $$M\left(H\right)=Ms[\coth\left(\mu HkBT\right)]$$

where *M*(*H*) is the magnetization as a function of the applied field (emu/cm^3^), Ms is the saturation magnetization (emu/cm^3^), μ is the average magnetic moment per nanoparticle (emu), H is the applied magnetic field (Oe), kB is the Boltzmann constant (1.3806 × 10^−16^ erg/K), and T is the absolute temperature (300 K). Since the experimental data were obtained in mass magnetization units (emu/g), the data were converted to volumetric magnetization (emu/cm^3^) using the density of the magnetite via the following equation:

(2) $$Mv\left(H\right)=\rho \cdot Mm(H)$$

where *Mv*(*H*) is the volumetric magnetization (emu/cm^3^), Mm(H) is the mass magnetization (emu/g), and ρ is the density of the magnetite (5.2 g/cm^3^). The average magnetic moment per nanoparticle was related to its volume, assuming spherical geometry, as follows:

(3) $$\mu =M_{sv}\cdot V=\frac{M_{sv}\cdot \pi d^{3}}{6}$$

where M_sv_ is the volumetric saturation magnetization (emu/cm^3^), V is the nanoparticle volume (cm^3^), and d is the average nanoparticle diameter (cm). For magnetite, the typical bulk saturation magnetization was considered as M_sv_ ≈ 480 emu/cm^3^ [45].

The experimental mass magnetization data (emu/g) were converted to volumetric units (emu/cm^3^) using the known density of magnetite. Nonlinear least-squares fitting was applied to fit the experimental data to the Langevin function. The adjustable parameters were the saturation magnetization M_sm_ and the average magnetic moment per nanoparticle μ. The fitting procedure was implemented using a computational environment, OriginLab. Upon determination of μ, the average nanoparticle diameter d was calculated assuming spherical shape, via the following equation:

(4) $$d=\frac{\left(6\mu \pi M_{sv}\right)}{3d}\;$$

All variables were appropriately converted to ensure dimensional consistency. The fitted volumetric saturation magnetization M_sv_ was then reconverted to mass magnetization units (emu/g) to allow direct comparison with experimental data, using the following equation:

(5) $$M_{sm}=M_{sv}\rho$$

where M_sm_ is the saturation mass magnetization (emu/g). The quality of the fitting was assessed through the coefficient of determination (R2R^2^) and residual analysis. The estimated average nanoparticle diameter was compared with values reported in literature for magnetite nanoparticles exhibiting superparamagnetic behavior. The obtained saturation magnetization was also compared with theoretical expectations for nanoscale magnetite, considering possible surface and finite-size effects [46].

## 3. Results and Discussion

**Figure 1:**
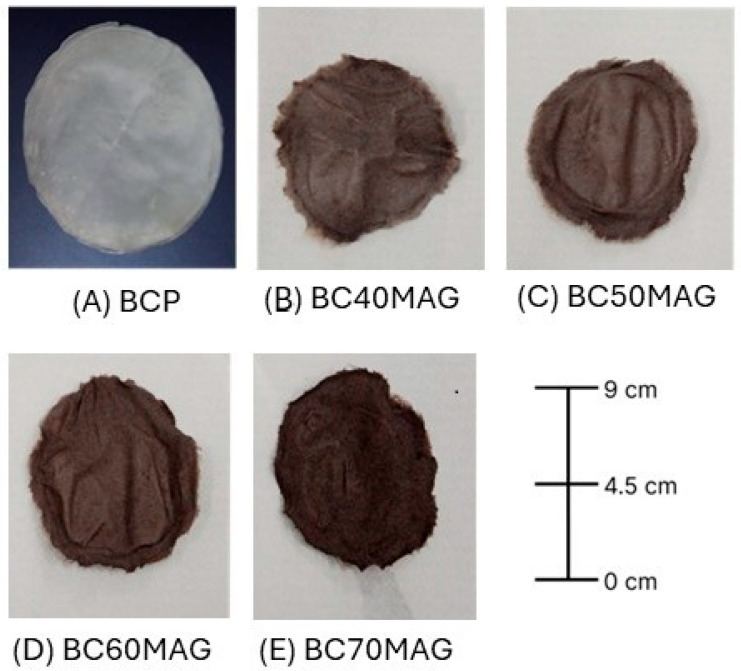
Bacterial cellulose biomembranes. (**A**) BCP = processed and dried BC in film form; (**B**) BC40MAG; (**C**) BC50MAG; (**D**) BC60MAG; (**E**) BC70MAG.

The BC biomembranes exhibited a uniform visual appearance, with a dark coloration corresponding to the amount of magnetite added. Figure 1 shows the aspect of the materials obtained.

### 3.1. X-Ray Diffraction (XRD)

**Figure 2:**
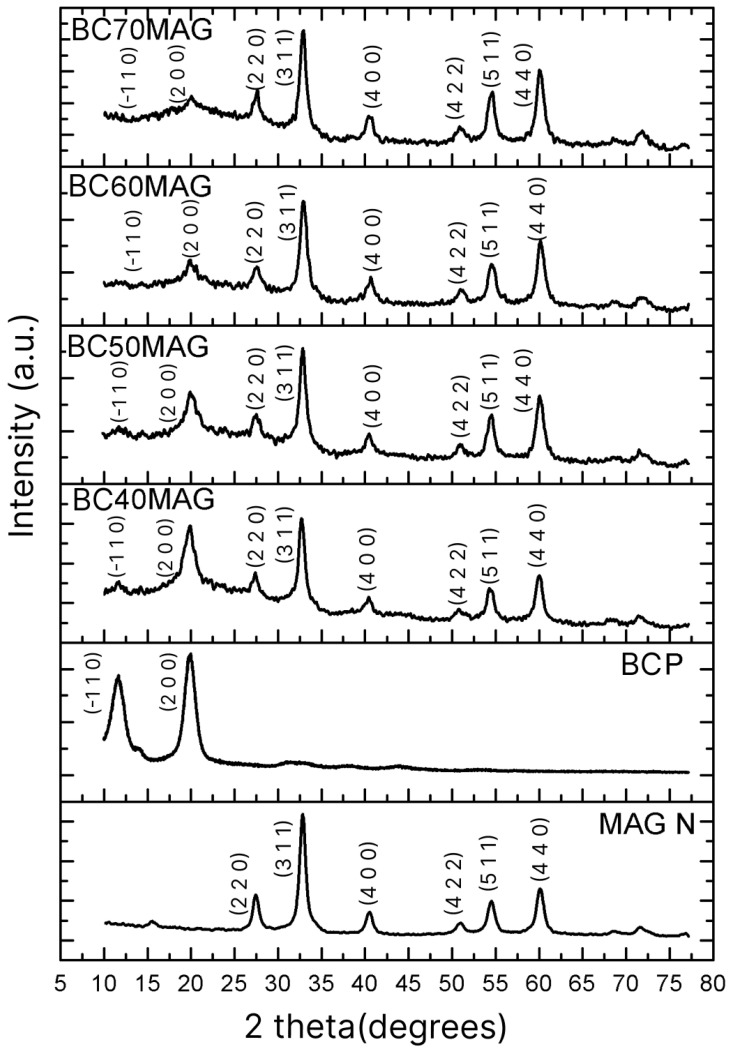
XRD spectra of the produced materials: BC70MAG, BC60MAG, BC50MAG, BC40MAG, BCP, and MAG N; MAG N = magnetite nanoparticles.

According to the XRD spectra shown in Figure 2, the samples exhibited the crystalline planes (−1 1 0) and (2 0 0), characteristic of cellulose [37,47], with the planes (2 2 0), (1 0 4), (3 1 1), (4 0 0), (4 2 2), (5 1 1), and (4 4 0) corresponding to magnetite [25,48,49,50]. The magnetite produced through the optimized methodology exhibited crystallites with an average diameter (6.96 nm), significantly smaller than those prepared by Souza et al. [29], using ex situ coprecipitation and processing (20, 20 nm). Table 1 provides a comparative overview of the crystallite sizes and crystallinity of the Fe_3_O_4_ particles formed in this study and those reported by Souza et al. [29], who performed ex situ synthesis without optimizations.

Such a reduction in diameter can be attributed to the enhanced control of parameters involved in the production of Fe_3_O_4_, as discussed previously. Namely, the adjusted ratio of iron ions, increased stirring, and sample purification may have significantly impacted the final nanoparticle size [30,31,32,33].

With this average diameter, the new magnetite nanoparticles can be classified as superparamagnetic, since, according to Giradet et al. [51], the critical diameter for Fe_3_O_4_ particles exhibiting such behavior is 20 ± 5 nm. The crystallinity of the nanoparticles also differed from that of the previous study. Souza et al. [29] reported nanoparticles with a crystallinity of 43%, whereas in the present study, a crystallinity of 60.5% was achieved, thanks to the optimization of the synthesis process. This increase in crystallinity indicates that the particles obtained under optimized conditions have a higher degree of molecular organization, with more aligned and regular structural patterns, resulting in higher stability, lower water absorption, and consequently a reduced risk of conversion to maghemite [52].

### 3.2. Fourier-Transform Infrared (FTIR) Spectrometry

**Figure 3:**
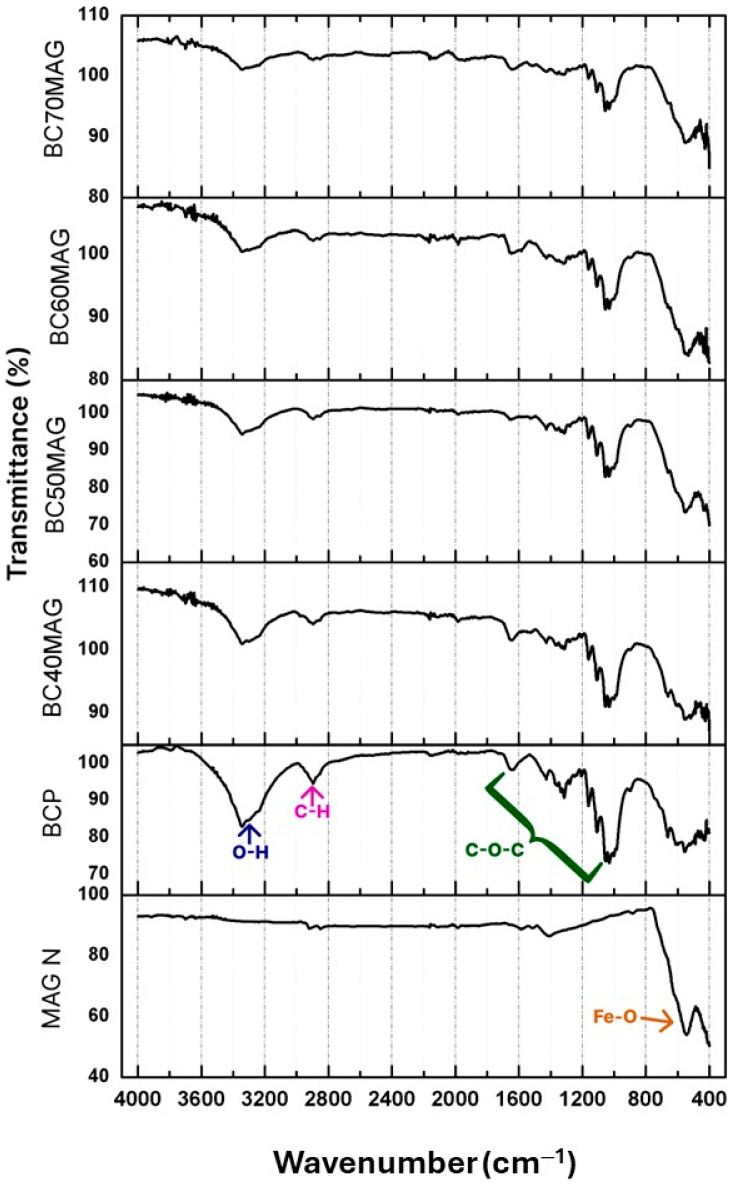
FTIR spectra of the produced materials, with the demarcation of its main peaks: BCP, BC70MAG, BC60MAG, BC50MAG, BC40MAG, and MAG N.

As shown in Figure 3, the samples exhibited vibrational peaks characteristic of the chemical bonds present in cellulose at wavenumbers of 2914, 1415, 1080, 839, and 3122 cm^−1^, corresponding to the asymmetric stretching of C-H, CH_2_, C-O, C-C, and O-H [17,49], respectively. Additionally, vibrational peaks related to Fe-O and Fe-O-C bonds were identified between 586 and 598 cm^−1^, confirming the presence of magnetite and its association with BC. Finally, the clear increase in the transmittance of iron-related peaks observed in the BC60MAG and BC70MAG samples can be ascribed to the increased amount of incorporated Fe_3_O_4_.

### 3.3. Scanning Electron Microscopy (SEM)

**Figure 4:**
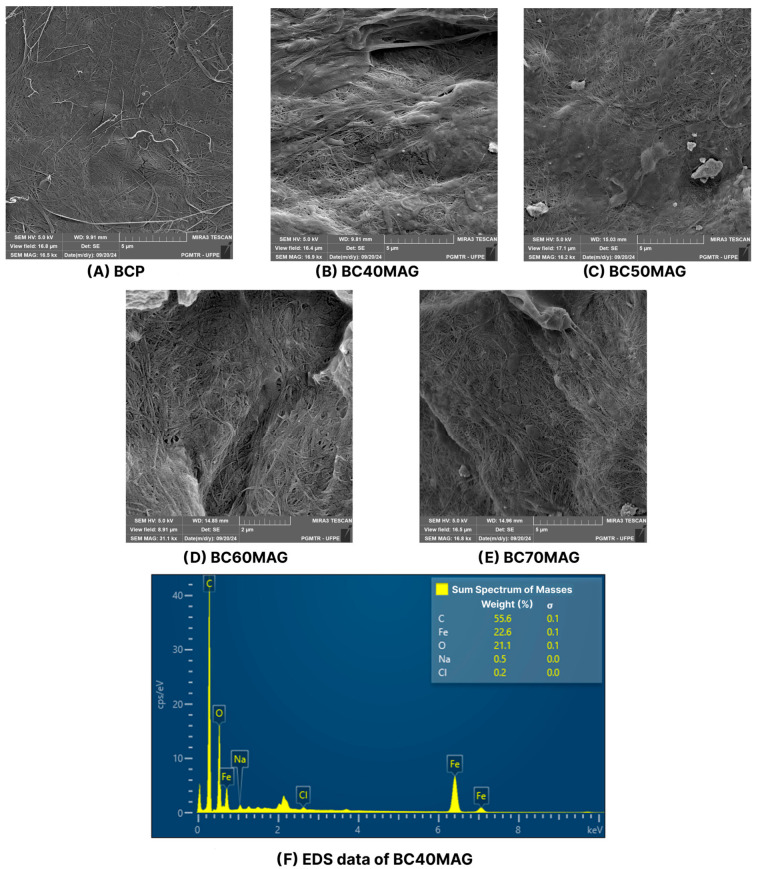
SEM images of the produced materials, with the magnifications of: BCP = 16.5 kx (**A**); BC40MAG = 16.9 kx (**B**); BC50MAG = 16.2 kx (**C**); BC60MAG = 31.1 kx (**D**); and BC70MAG = 16.8 kx (**E**). EDS of the CB40MAG sample, with the percentage of iron (**F**).

According to the SEM images (Figure 4), the samples had surfaces on which BC fibers and magnetite nanoparticles were visible. The arrangement of compounds in the samples was similar to that observed by Chaabane et al. [17] and Chanthiwong et al. [53] in cellulose nanofibers, whose coating with nanoparticles made them thicker.

The fibers obtained from crushed BC membranes appeared to be reorganized in conjunction with the added nanoparticles, forming a more homogeneous surface with few agglomeration points (Figure 4). Figure 4A shows the BC fibers in the BCP sample, without the nanoparticles, that coated the fibers in the other samples, as shown in Figure 4B–E. This characteristic is similar to that recently observed by Souza et al. [29] for a magnetic BC produced using the ex situ coprecipitation method, which, however, exhibited a surface richer in Fe_3_O_4_ particles, likely due to the higher incorporation of nanoparticles into the BC (82.92%) and the larger average size of its crystallites (31.03 nm).

The EDS graph presented in Figure 4F indicates a strong presence of iron in the composition of the samples, as well as residual reagents in smaller quantities, such as sodium and chlorine, likely derived from the reagents used during the production process.

### 3.4. Vibrating Sample Magnetometry (VSM)

**Figure 5:**
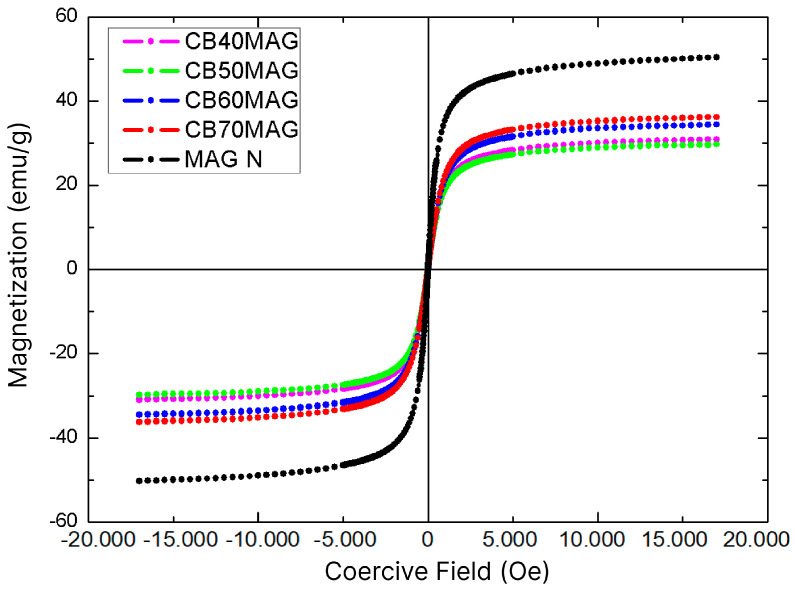
VSM graphs of the produced materials: BC70MAG, BC60MAG, BC50MAG, and MAG N.

The hysteresis loops of specimens measured by VSM are illustrated in Figure 5, and their respective saturation magnetization (Ms) values, obtained through the Langevin curve, are listed in Table 2. For comparison purposes, Table 2 also presents the magnetic properties corresponding to the pure magnetite samples and the BC-based materials with magnetite, as reported in the study by Souza et al. [29], which were also synthesized using the ex situ coprecipitation method with BC processing, albeit without the optimizations.

The crystallinity of the particles is an important factor for the saturation magnetization (Ms), as crystalline structures with more irregularities can disrupt the alignment of magnetic moments, while a greater structural organization allows more magnetic moments to effectively contribute to an increase in Ms. Low crystallinity may also indicate defects in the crystal structure, altering the magnetic interaction between Fe^2+^ and Fe^3+^ ions in the inverse spinel structure of magnetite [42,54,55]. Thus, due to the crystallite size and the high purity of the obtained magnetite, the samples achieved higher Ms values than those recently obtained by Souza et al. [29], even with lower percentages of magnetite in their compositions.

Particularly, the Ms of magnetite synthesized in this study under optimized conditions (36.2 emu/g) and non-optimized conditions (29.7 emu/g) were about 113% and 75% higher than the highest value reported by those authors for magnetic BC prepared by in situ coprecipitation and processing (17 emu/g). Moreover, the obtained samples did not exhibit a significant coercive field, demonstrating superparamagnetic behavior [53,56] with a faster response to external magnetic fields, an ideal characteristic for sensors and biosensors [3,57,58], as these devices rely on quick responses to magnetic oscillations, as well as for electromagnetic resonance contrast agents [59] and drug delivery systems [25,60], which require materials that do not exhibit magnetic remanence, a feature that could hinder these applications [22]. This contrasts with the particles obtained through the same synthetic route but without optimizations, as reported in the previous study [29], where the material exhibited a significant coercive field.

Based on the relationship between the graphs shown in Figure 5 and the Langevin curve, it was also possible to estimate the radius of the Fe_3_O_4_ nanoparticles in each sample. All values were close to 5.3 nm, which was relatively consistent with the crystallite size obtained from XRD analysis using the Scherrer equation, a typical size for a superparamagnetic magnetite nanoparticle. Similar results have been reported in literature; in Khanam et al. [61], the authors synthesized magnetite nanoparticles using the coprecipitation method and observed an average particle size of 6.8 nm, along with a saturation magnetization of 49.88 emu/g—values comparable to those found in the present study. The particle size values obtained were smaller than those reported by Osipov et al. [62], who synthesized the nanoparticles using a laser-based method. However, they were similar to the superparamagnetic particles produced by Darbandi et al. [63], who employed microemulsion techniques. Chanthiwong et al. [50] and Sriplai et al. [56] also produced superparamagnetic magnetite particles with sizes and saturation magnetization values similar to those obtained in the present study.

## 4. Conclusions

Magnetic materials based on BC were produced with different concentrations of magnetite. The results confirmed that, through modified parameters such as the molar ratio of reagents, stirring, and purification, it was possible to optimize the methodology to obtain an innovative biomaterial containing superparamagnetic magnetite particles. The obtained magnetite nanoparticles exhibited crystallites with reduced average diameter, higher crystallinity, and higher saturation magnetization than those obtained in previous studies conducted in our laboratories.

The samples with a higher percentage of incorporated magnetite (BCMAG60 and BCMAG70) showed higher saturation magnetization, indicating greater sensitivity to external magnetic fields. The produced biomaterials can be applied in areas such as electronics, sensors, EMI shielding, and enzyme immobilization, among others.

## Figures and Tables

**Table 1:** Comparative values of magnetite made by the coprecipitation method obtained in this work and in the study by Souza et al. [18].

Magnetite	Crystallinity (%)	Crystallite Size (nm)
In this study	60.5	6.96
Souza et al. [29]	43.0	20.20

**Table 2:** Values of saturation magnetization (Ms) for each produced sample and the others found in the study by Souza et al. [29].

Sample	Magnetite Concentration (%)	Saturation Magnetization (emu/g)	Coercive Field (Oe)
Fe_3_O_4_ (MAG N)	100	50.0	≈0
Fe_3_O_4_ [29]	100	29.0	87, 57
BCP-COMAG [29]	82, 92	10.0	22, 07
BC40MAG	40	29.6	≈0
BC50MAG	50	30.7	≈0
BC60MAG	60	34.4	≈0
BC70MAG	70	36.2	≈0

## Data Availability

The original contributions presented in this study are included in the article. Further inquiries can be directed to the corresponding author.
